# Computer-Supported Collaborative Design of Standardized Clinical Cases: Algorithm Development and Validation

**DOI:** 10.2196/45315

**Published:** 2023-09-19

**Authors:** Sergio Guinez-Molinos, Félix Buendía-García, José-Luis Sierra-Rodríguez, Joaquín Gayoso-Cabada, Jaime González-Díaz

**Affiliations:** 1 School of Medicine, Universidad de Talca, Talca, Chile; 2 Escuela Técnica Superior de Ingeniería Informática, Universitat Politècnica de Valencia, Valencia, Spain; 3 Facultad de Informática, Universidad Complutense de Madrid, Madrid, Spain; 4 Escuela Técnica Superior de Ingeniería de Sistemas Informáticos, Universidad Politécnica de Madrid, Madrid, Spain

**Keywords:** collaborative learning, interoperability, case-based learning, standards, clinical cases, collaborative clinical cases

## Abstract

**Background:**

The creation of computer-supported collaborative clinical cases is an area of educational research that has been widely studied. However, the reuse of cases and their sharing with other platforms is a problem, as it encapsulates knowledge in isolated platforms without interoperability. This paper proposed a workflow ecosystem for the collaborative design and distribution of clinical cases through web-based computing platforms that (1) allow medical students to create clinical cases collaboratively in a dedicated environment; (2) make it possible to export these clinical cases in terms of the Health Level 7 (HL7) Fast Healthcare Interoperability Resources (FHIR) interoperability standard; (3) provide support to transform imported cases into learning object repositories; and (4) use e-learning standards (eg, Instructional Management Systems Content Packaging [IMS-CP] or Sharable Content Object Reference Model [SCORM]) to incorporate this content into widely-used learning management systems (LMSs), letting medical students democratize a valuable knowledge that would otherwise be confined within proprietary platforms.

**Objective:**

This study aimed to demonstrate the feasibility of developing a workflow ecosystem based on IT platforms to enable the collaborative creation, export, and deployment of clinical cases.

**Methods:**

The ecosystem infrastructure for computer-supported collaborative design of standardized clinical cases consists of three platforms: (1) Mosaico, a platform used in the design of clinical cases; (2) Clavy, a tool for the flexible management of learning object repositories, which is used to orchestrate the transformation and processing of these clinical cases; and (3) Moodle, an LMS that is geared toward publishing the processed clinical cases and delivering their course deployment stages in IMS-CP or SCORM format. The generation of cases in Mosaico is exported in the HL7 FHIR interoperability standard to Clavy, which is then responsible for creating and deploying a learning object in Moodle.

**Results:**

The main result was an interoperable ecosystem that demonstrates the feasibility of automating the stages of collaborative clinical case creation, export through HL7 FHIR standards, and deployment in an LMS. This ecosystem enables the generation of IMS-CPs associated with the original Mosaico clinical cases that can be deployed in conventional third-party LMSs, thus allowing the democratization and sharing of clinical cases to different platforms in standard and interoperable formats.

**Conclusions:**

In this paper, we proposed, implemented, and demonstrated the feasibility of developing a standards-based workflow that interoperates multiple platforms with heterogeneous technologies to create, transform, and deploy clinical cases on the web. This achieves the objective of transforming the created cases into a platform for web-based deployment in an LMS.

## Introduction

In March 2020, the World Health Organization (WHO) declared COVID-19 a pandemic [1], directly affecting traditional teaching in medical schools worldwide [2,3]. As a result, at the beginning of April 2020, classroom training activities were suspended at all universities, and students’ clinical training was disrupted by the collapse of public and private hospitals [4]. This situation has prompted new teaching strategies for medical students, who could not attend traditional clinical rotations, representing one of the main challenges in this educational context [5]. In this sense, COVID-19 triggered alternative IT-based medical education methodologies, with synchronous and asynchronous mechanisms to be deployed in web-based learning environments [6]. Afterward, web-based medical education’s need for content production has quickly become apparent.

Computer-supported, didactic medical content production in web-based environments demands innovative platforms that enable collaboration among medical students and instructors. Mosaico is an example of a web platform [7] that allows users to design, perform, and assess collaborative clinical scenarios for medical students. Similar platforms have been developed to foster collaborative learning for medical education. For example, Osmosis [8] is a web and mobile learning platform that provides access to multiple clinical questions and explanations for medical student self-assessments. Wikis have also been used as collaborative platforms to build and curate didactic content in graduate medical education [9]. Vicente et al [10] created a platform to enable collaborative learning in the context of the One Health initiative, which aimed to elaborate a corpus of graduate courses or modules in multiple health care disciplines. In addition, Alicanto [11] is a web-based collaboration platform for health care professionals that helps them to share educational resources or discuss clinical cases.

The main advantage of these platforms is to support the learning of groups of students who participate in collaborative activities oriented to generating clinical cases, supporting a didactic approach based on the collaborative design of clinical cases, whose educational value has been widely demonstrated. However, its main shortcoming is that the contents produced are usually oriented to self-consumption within the tools. This restriction limits the reuse of content by not having the ability to export to other platforms, such as conventional learning management systems (LMSs) [12].

Therefore, considering the multiple challenges imposed by the COVID-19 pandemic and the adequacy of practical teaching, it becomes evident that support technology for the teaching-learning processes must allow the export and sharing of the material generated. Thus, we must expand the frontiers of knowledge and open up the consumption of clinical cases created by tools at all levels, starting with LMS platforms. Otherwise, content reuse in contexts other than production tools will be seriously hampered. This paper aimed to address this problem.

We proposed using Mosaico as a platform for the computer-supported collaborative design of educational clinical cases through collaborative clinical simulation [13,14]. To extend and democratize the content generated, Mosaico was extended with an interoperability add-on implemented with an internationally accepted standard, Health Level 7 (HL7) Fast Healthcare Interoperability Resources (FHIR) [15], which allows the designed clinical cases to be exported to a wide variety of third-party platforms. In addition, it allowed us to connect Mosaico with Clavy [16], a tool for managing learning object repositories that enables the transformation into a wide variety of formats and has demonstrated its applicability to the generation of medical learning content from preexisting medical collections. Using Clavy’s transformation capabilities, clinical cases retrieved from Mosaico and rendered in HL7 FHIR can be transformed into standardized learning content formats that can be deployed in web-based LMSs such as Moodle or Blackboard, which ultimately enabled us to meet the goal of exporting collaboratively designed Mosaico clinical cases to widely used conventional web-based learning platforms. In this sense, we linked the generation of content with its deployment, sharing it with e-learning environments and extending its application and use by medical students, who can access and share clinical content in a standardized way.

To demonstrate the feasibility of the developed ecosystem, a clinical case of chest pain that considers the whole workflow (collaborative design, transformation, deployment, and consumption) is presented.

## Methods

### Overview

The collaborative design of clinical cases, created by medical students through a computer-based platform, supports integrating what has been learned and generating knowledge consumed by other students.

**Figure 1.**
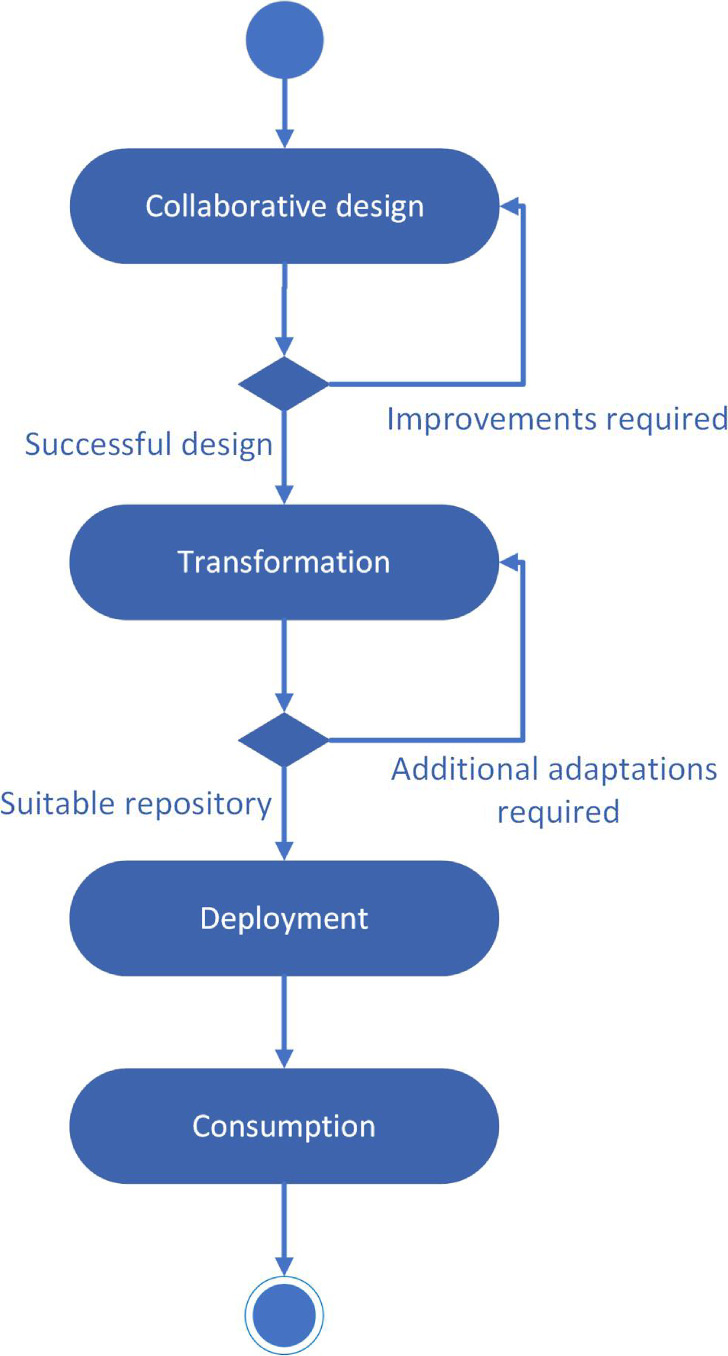
Activity diagram for the collaborative production and exploitation of clinical cases.

The activity diagram of Figure 1 depicts our approach to case-based learning in clinical undergraduate programs. According to this diagram, the approach comprises the following activities:

- *Collaborative design*: In this activity, while facilitated by a reputed instructor, a community of advanced students is involved in the collaborative production of clinical cases. This activity is carried out until the community reaches a clinical case that the instructor judges to be satisfactory.
- *Transformation*: In this activity, the collaboratively produced clinical cases are transformed by instructors into standardized learning packages. To do this, instructors can carry out different types of transformations, which take a given representation of clinical cases as input and generate refined representations as output. These transformations can be carried out automatically, semiautomatically (with instructors configuring some of the automatic processes involved), or manually (with instructors editing content with appropriate editing tools). The process evolves, in general, through several transformation steps until a suitable representation is reached, which is then directly exported into standardized e-learning packages.
- *Deployment*: In this activity, instructors can publish the e-learning packages resulting from the successive transformations into LMSs that support the e-learning standards used to code these content packages.
- *Consumption*: In this activity, less advanced students can take advantage of the conventional e-learning courses generated based on the clinical cases produced in the initial collaborative environment.

Consequently, learning occurs in two different scenarios:

1. Learning takes place during the collaborative design of clinical cases, which is oriented to the community of advanced learners mediated by the instructor (*Collaborative design* activity).
2. Once clinical cases are transformed by instructors into standardized learning packages, more conventional learning takes place based on content published in a standard LMS (*Consumption* activity).

**Figure 2.**
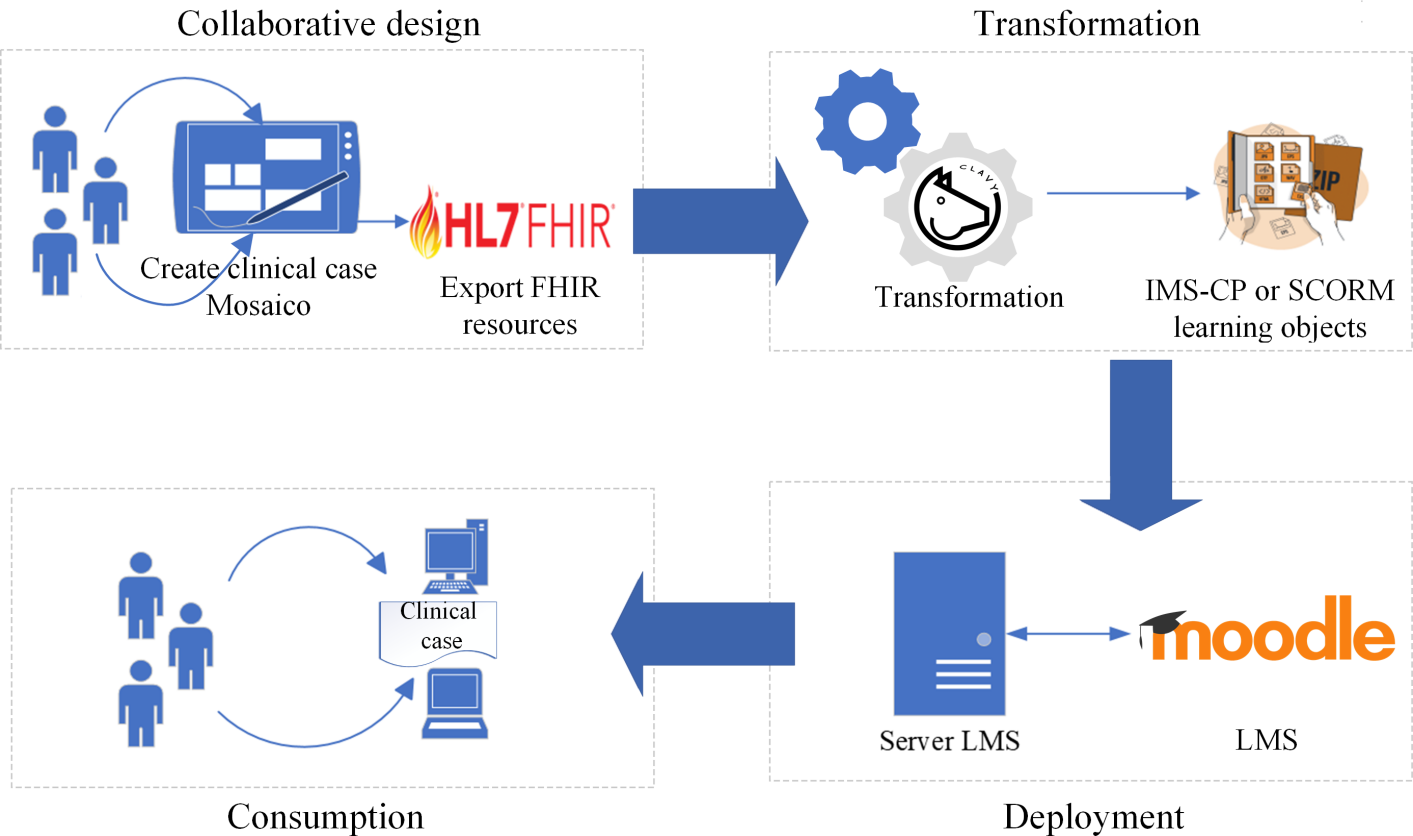
Workflow of the collaborative creation of a clinical case, interoperable with Health Level 7 (HL7) Fast Healthcare Interoperability Resources (FHIR) and packaged as Instructional Management Systems Content Packaging (IMS-CP) or Sharable Content Object Reference Model (SCORM) standard learning objects to be distributed in any learning management system (LMS) that reads these standards.

In this context, we designed and developed a workflow ecosystem based on the collaborative cocreation of clinical cases, supported by technological platforms and global standards that allow for the standardization, export, and distribution of clinical cases for open and democratic consumption. This ecosystem, which is illustrated in Figure 2, integrates the following platforms:

- Mosaico: The platform is used in computer-supported collaborative design of clinical cases [7].
- Clavy: A tool for the flexible management of learning object repositories that is used to orchestrate the transformation stage [16].
- Moodle: The LMS orchestrates the publishing and course consumption stages in the Instructional Management Systems Content Packaging (IMS-CP) or Sharable Content Object Reference Model (SCORM) format [17].

### Mosaico for Designing, Performing, and Assessing Collaborative Clinical Cases

**Figure 3.**
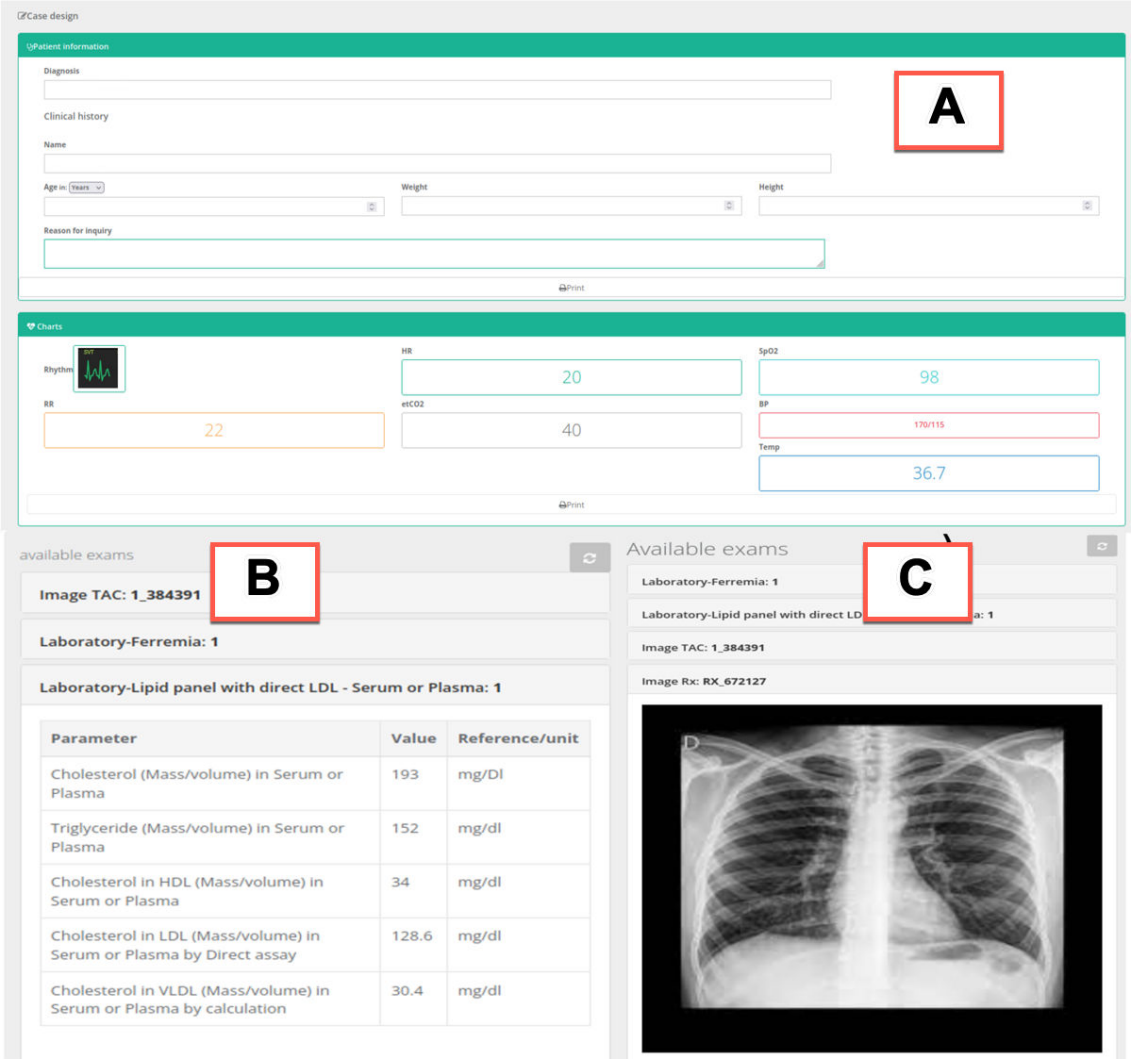
Screenshot of the case design form provided by Mosaico, separated into the following sections: (A) patient information and vital signs, (**B**) patient’s laboratory exams, and (C) images and videos (in this case, an x-ray of the thorax). HDL: high-density lipoprotein; LDL: low-density lipoprotein; VLDL: very low–density lipoprotein.

For the collaborative design of a clinical case, Mosaico provides standardized templates with all the information required. Students would write down relevant information about the clinical case, including age, sex, weight, height, physical exam, vital signs, laboratory tests, images, or videos (see Figure 3).

The web platform allows clinical cases to be generated, guided, and monitored; however, it stores them in a relational database. This does not allow records to be extracted and shared across heterogeneous technologies. In addition, Mosaico’s lack of interoperability features limits the platform’s use by forcing all institutions to use the same technology or make ad hoc integrations if they wish to benefit from the portfolio of clinical cases created.

### Extraction and Standardization of Clinical Cases

To provide Mosaico with interoperability capabilities, we proposed including a module that allows the transformation and export of clinical cases into a universal format—one that any other platform can easily read.

This module incorporates an interoperability layer designed and implemented through REST application programming interfaces [18] with HL7 FHIR [19] that offers clinical knowledge representation based on international standards. For this purpose, and according to the case design form provided by Mosaico (Figure 3), a set of HL7 FHIR resources would contain all the standardized clinical case information, making it easily exportable.

### Transformation of Interoperable Clinical Cases With Clavy

Clavy is a learning object repository management platform based on formal grammars. Each Clavy repository comprises (1) a set of object schemata, which describes the structures of the different objects using formal grammars, and (2) the learning objects themselves. In addition, the platform fully supports Extract-Transform-Load (ETL) workflows with user-defined plug-ins and advanced editing facilities.

**Figure 4.**
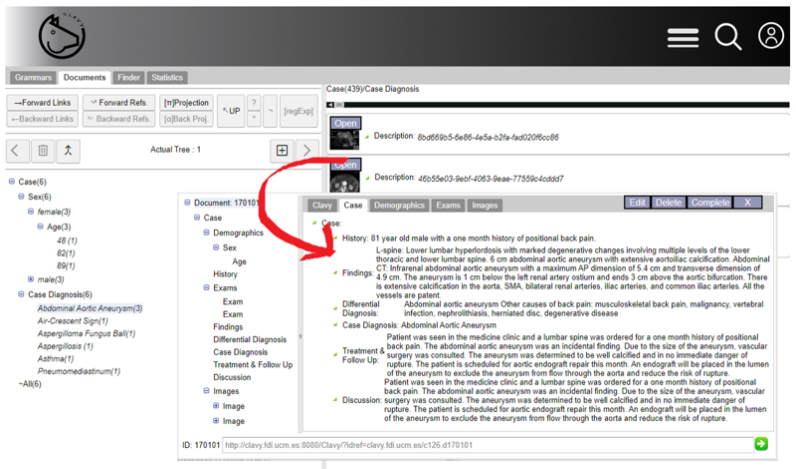
Clavy snapshot of a medical repository (navigation tree, learning object collection, and a particular learning object).

The tools integrated by Clavy can be accessed on the web and provide user-friendly interfaces to manage the repositories. Using these tools, domain experts without programming knowledge can use different transformations to reorganize the repository’s structure, remove irrelevant information, and add new objects to this repository. For example, Figure 4 shows a screenshot that displays part of a specialized repository in the clinical domain, making it appear as a part of a schema-guided navigation tree, a part of the learning object collection, and a fragment of a particular learning object.

## Results

### Clinical Case

We applied our workflow ecosystem to generate standardized e-learning content for a relevant clinical case. The clinical case to show the proposed approach was related to a patient who consults for chest pain. The case was designed while considering all the clinical backgrounds necessary for its representation through a simulated patient. Furthermore, it was important to consider all the antecedents since the case will later be packaged and distributed to an LMS environment so that other students can consult it as study material.

### Collaborative Chest Pain Case Development

Fourth-year medical students, along with an academic instructor from the University of Talca in Chile, developed the case in Mosaico by considering the following scenario:

A 58-year-old male patient with a history of arterial hypertension (treated with 20 mg of enalapril every 12 h, which he takes irregularly), who works as an engineer 8 hours per day in an office, consults for oppressive chest pain in the precordial region, which is radiating to the neck and jaw, appearing at rest, and accompanied by coldness and cutaneous pallor that accentuated approximately 8 hours ago. He went to the hospital, was evaluated, and presented the following vital signs:

- Blood pressure: 170/115 mm Hg
- Heart rate: 95 beats per minute
- Respiratory rate: 22 respirations per minute
- Oxygen saturation: 98%
- Temperature: 36.7 °C

Moreover, the following complementary tests were incorporated: laboratory exams, chest x-rays, and an electrocardiogram taken in the hospital.

**Figure 5.**
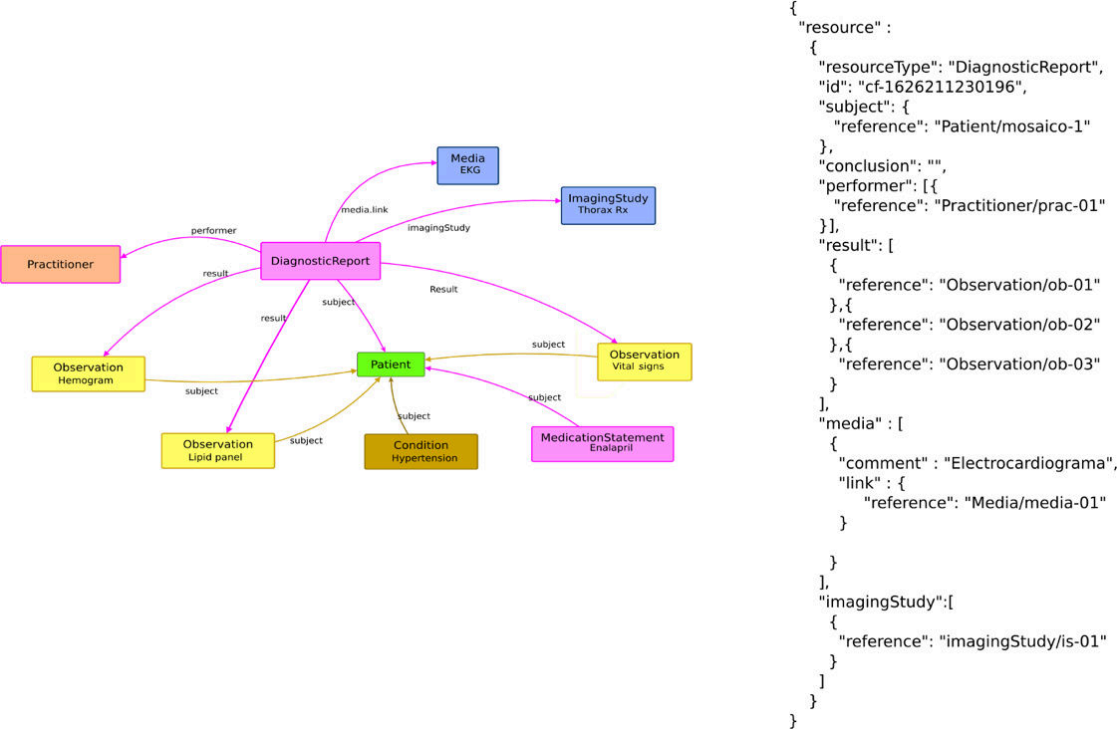
Chest pain case standardized with Health Level 7 (HL7) Fast Healthcare Interoperability Resources (FHIR) resources and respective JSON structure. EKG: electrocardiogram; Rx: prescription.

The case was created in Mosaico (see Figure 3) and stored in a MYSQL relational database; all data were then exported as HL7 FHIR resources. Figure 5 shows the resources involved, using a DiagnosticReport resource as the backbone of the case.

### Use of Clavy to Generate Standardized e-Learning Content

**Figure 6.**
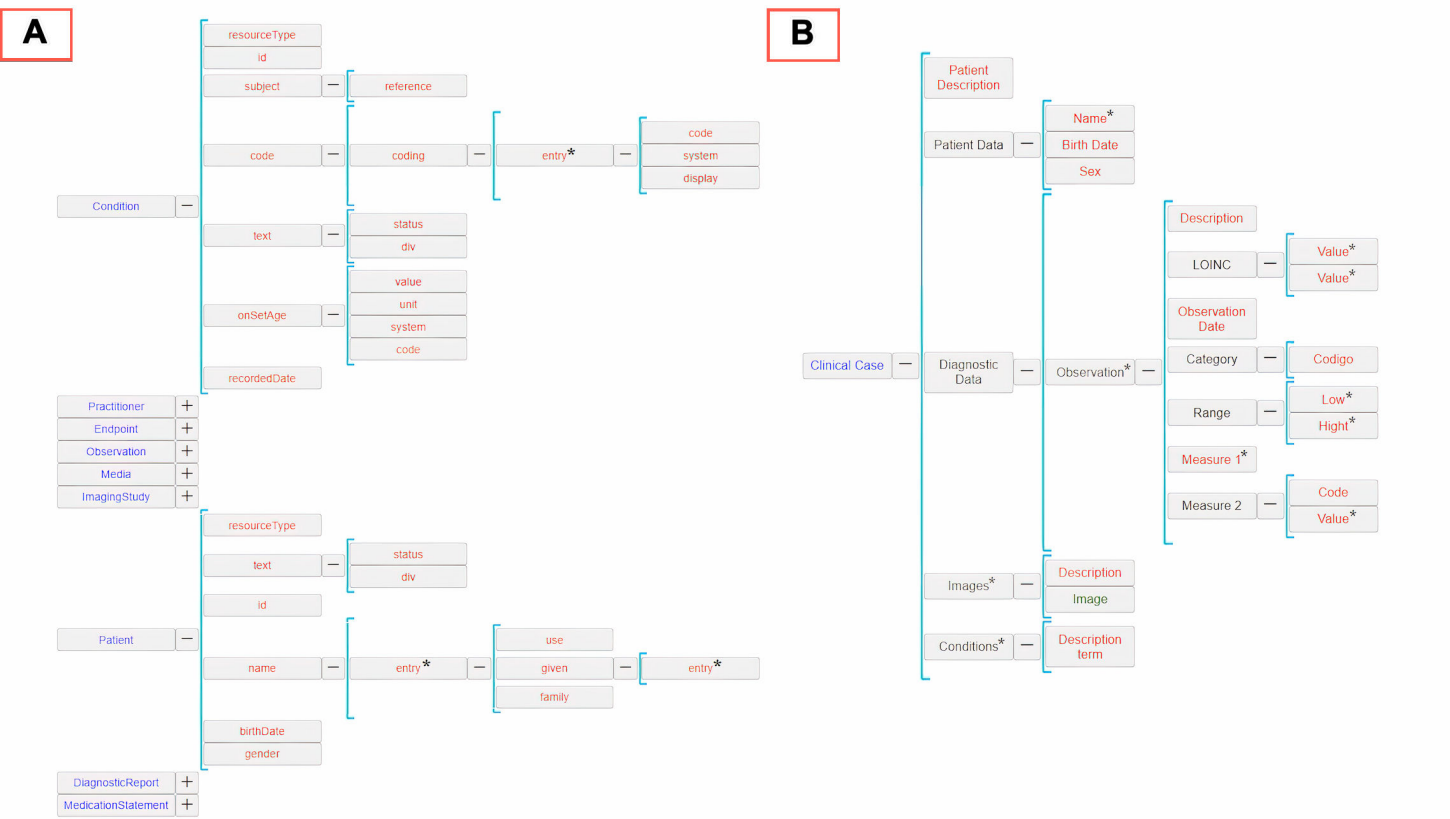
(A) Clavy schema for the Health Level 7 (HL7) Fast Healthcare Interoperability Resources (FHIR) entities generated by Mosaico and (**B**) the schema for the repository that results after the transformation and editing steps.

Clavy was used to create an ETL pipeline to extract and transform the information created in Mosaico; this information could then be readily exported into IMS-CPs that could be deployed in an LMS, such as Moodle or Blackboard. In this way:

- The proposed pipeline begins with a generic JSON import plug-in, which lets Clavy ingest the Mosaico-generated JSON files encoding the HL7 FHIR entities associated with the clinical cases. As a result, a first repository is generated with (1) an object schema for each of the FHIR entity types ingested and (2) an object for each FHIR resource collected. Figure 6A shows a part of the Clavy schemata for the repository generated.
- Next, several automatic transformations are applied. The first transformation acts on the repository extracted by the JSON import plug-in to incorporate the FHIR semantics by making the relationships in FHIR instances explicit. The second transformation, in turn, enriches the repository with Systematized Nomenclature of Medicine (SNOMED) data. For this purpose, it detects SNOMED terms in learning objects, adds the SNOMED terms discovered to the repository as new objects, and links the FHIR entities with these new SNOMED objects as needed. Finally, the third transformation makes it possible to aggregate a set of interrelated FHIR entities into a single learning object. For this purpose, an entity that will act as the main one is selected, and a closure process is carried out to convert the nonhierarchical relationships into hierarchical ones. In this experience, the central entity chosen was the *Patient*.
- Finally, once a learning object repository focused on a particular kind of FHIR entity (*Patient*, in this case) is generated, its schema can be edited by using the Clavy repository editing facilities to accommodate all this information to the instructors’ needs (in our case, the generation of standardized e-learning content). This experience allowed us to remove useless and irrelevant categories from the repository schema and, therefore, from the learning objects. Figure 6B illustrates the schema resulting from this editing step. Notice that, on the one hand, as produced by the previous transformation step, this schema characterizes only one type of learning object (*Patient*), aggregating all the relevant information in all the interrelated FHIR entities. However, on the other hand, many irrelevant FHIR information items from an educational point of view have been safely removed in this editing step.

### Deployment of IMS-CP in LMS Environments

**Figure 7.**
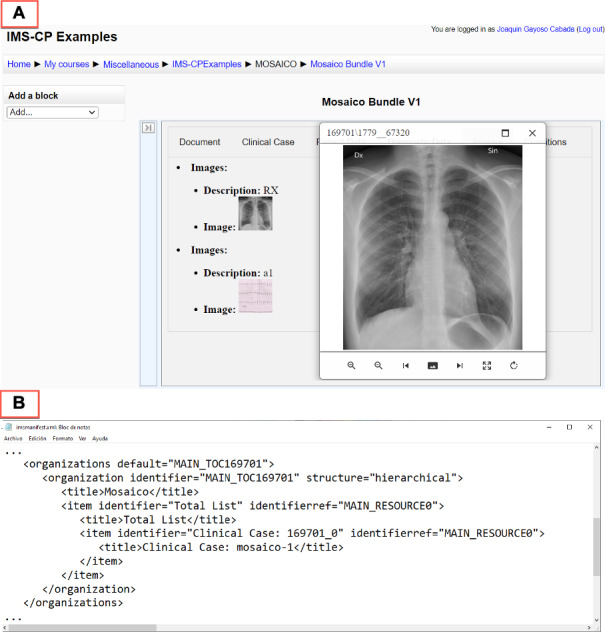
(**A) **A learning object associated with the Mosaico clinical case imported into Moodle’s platform as an Instructional Management Systems Content Package (IMS-CP) and (**B**) an excerpt of the IMS-CP manifest file.

Once the information has been transformed into a structure that suits the instructors’ needs, a suitable Clavy export plug-in can be applied to the resulting repository to package the clinical cases into standardized learning packages that can be loaded into conventional LMSs. In this experience, the clinical case was exported as IMS-CPs and incorporated into Moodle (see Figure 7A). Figure 7B shows an IMS-CP manifest file segment associated with the original Mosaico clinical case.

## Discussion

### Principal Findings

In this paper, we identified four key elements to enable the effective use of clinical cases in e-learning environments: (1) the effective authoring of cases, (2) the interoperability of authoring tools, (3) the transformation of cases into standard e-learning content packages, and (4) the publication of content packages on widely used e-learning platforms. For this purpose, we proposed a workflow that consistently addresses each of these critical aspects by forming a technology ecosystem supported by (1) the Mosaico collaborative clinical case authoring tool, which supports students and teachers in health care careers with the collaborative design of clinical cases; (2) the HL7 FHIR clinical interoperability standard, which makes it possible to expose relevant information from the clinical cases to third-party tools; (3) the Clavy learning object repository management tool, which allows the transformation of these clinical cases into standardized educational formats; and (4) the Moodle LMS, which makes it possible to deploy the resulting contents for their consumption.

The collaborative design supported by Mosaico [7] allows medical students to create clinical cases through standardized templates with all the information required and the possibility to integrate relevant knowledge from multiple sources. The case study regarding chest pain described in the previous section, in which Mosaico was successfully used to produce a complex clinical case by a group of advanced students under the supervision of a medical instructor, highlights the suitability of Mosaico in orchestrating this type of collaborative clinical case authoring. At this stage, it is essential to highlight Mosaico’s ability to allow the creation of clinical cases by groups of students, which can be shared and exchanged democratically within the academic community. An interesting question to discuss is the wide variety of medical scenarios that can be considered during the design of clinical cases. Since the clinical cases are created by students in the company of an academic instructor, they are never repeated. Each design session has a new case history being shared, regardless of the diagnosis.

Concerning interoperability issues, HL7 FHIR specifications or notations [20] standardize medical records by incorporating an interoperable format that increases the exchange potential of any type of health data source. As exemplified by the case study in the previous section, the interoperability layer added to Mosaico highlights the feasibility of representing complex educational and clinical cases in terms of the HL7 FHIR information models. In this regard, clinical cases created using tools such as Mosaico, represented internally in proprietary and tool-dependent formats, can be offered to third-party tools in standardized and open formats. As shown in the case study, this aspect is essential to facilitating the reuse of clinical cases in many contexts that are not necessarily anticipated. In the work described in this paper, FHIR has allowed the generation of information packages used to build digital medical repositories, which can be deployed in subsequent stages of the design process. A similar environment to the one proposed and aimed at collecting clinical case information based on FHIR notations can be found in the Public Health Automated Case Event Reporting platform [21], which uses laboratory results to extend the description of clinical cases.

Although the availability of clinical cases represented in an interoperable format, such as HL7 FHIR, facilitates their consumption by external tools, a substantial gap prevents their publication in e-learning environments. Indeed, as evidenced in the case study presented, at the syntactic level, the HL7 FHIR information packages produced by Mosaico consist of JSON files and additional associated resources (eg, clinical images). Meanwhile, they represent instances of the FHIR information models at the semantic level. In contrast, information publishable on e-learning platforms follows well-established e-learning standards (eg, IMS-CP or SCORM). Therefore, this is a domain transformation problem at the syntactic level (eg, the transformation of JSON documents into IMS-CP XML manifests) and at the semantic level (eg, the change of FHIR entity graphs into IMS-CP or SCORM organizations). As the case study shows, the Clavy platform demonstrated its ability to undertake the required transformation tasks. Indeed, in this paper, Clavy was able to convert the HL7 FHIR resources produced by Mosaico through automatic procedures, taking the resources as input and generating digital repositories to be edited and processed. In addition, during this transformation stage, the remarkable functionality of Clavy allowed text elements to be converted from Mosaico-based clinical cases into standard terms such as SNOMED Clinical Terms or Logical Observation Identifiers Names and Codes (LOINC), which adds value to the original case descriptions. The advantages of using semantic standardization terminologies have been reviewed in matters such as structured reporting [22] or health care process mining [23].

The final stage consists of generating standard educational specifications, which can be useful when spreading instructional content across diverse health care e-learning environments [24]. In this case, Clavy allowed the export of the clinical cases collected from Mosaico using standard formats such as IMS-CP or SCORM. Standard specifications have played a crucial role in applying e-learning technologies for medical education. Most of these specifications focus on the content sharing of health care learning resources that can be designed and developed collaboratively, as seen in the mEducator initiative [25]. The work presented in this paper is similar to this initiative, relying on learning objects and IMS-CP educational packages to deliver instructional clinical cases to medical students and health care practitioners. In addition, the role of technical standards in medical instructional settings has been reviewed as a part of recent digital innovations introduced in health care education and training [26]. These innovations boost the use of SCORM packages that allow e-learning developers to add enhanced interactive features and assessment procedures to the massive amount of medical knowledge available, which are traditionally stored as plain content items [27]. Moreover, they introduce the possibility of tracking user interactions with these content items to supervise their learning in clinical scenarios [28].

### Conclusions

In this paper, we proposed and implemented a workflow to deploy standard e-learning content, combining Mosaico, a tool for collaborative content authoring; HL7 FHIR, a clinical information interoperability standard; and Clavy, a platform for the flexible management of learning object repositories. Through a substantial case study, we demonstrated (1) how clinical cases collaboratively developed in Mosaico can be coded and exported in HL7 FHIR, (2) how this facilitates the reuse of these clinical cases with other tools, and (3) how cases exported in HL7 FHIR can be transformed into IMS-CP e-learning packages using a tool such as Clavy. As a result, we demonstrated the feasibility of deploying collaboratively created content using proprietary authoring tools in conventional open e-learning environments.

Having demonstrated the approach’s feasibility, we intend to apply it to other scenarios of collaborative production of medical e-learning content. Likewise, we want to better integrate the components that constitute the proposed workflow ecosystem to the end users transparently, thus increasing usability and the overall user experience.

## Abbreviations

ETL: Extract-Transform-Load
FHIR: Fast Healthcare Interoperability Resources
HL7: Health Level 7
IMS-CP: Instructional Management Systems Content Packaging
LMS: learning management system
LOINC: Logical Observation Identifiers Names and Codes
SCORM: Sharable Content Object Reference Model
SNOMED: Systematized Nomenclature of Medicine
WHO: World Health Organization
